# Signalling pathways involved in hypoxia‐induced renal fibrosis

**DOI:** 10.1111/jcmm.13060

**Published:** 2017-01-18

**Authors:** Minna Liu, Xiaoxuan Ning, Rong Li, Zhen Yang, Xiaoxia Yang, Shiren Sun, Qi Qian

**Affiliations:** ^1^ Department of NephrologyXijing HospitalFourth Military Medical UniversityXi'anShaanxiChina; ^2^ State Key Laboratory of Cancer BiologyFourth Military Medical UniversityXi'anShaanxiChina; ^3^ Department of GeriatricsXijing HospitalFourth Military Medical UniversityXi'anShaanxiChina; ^4^ Department of MedicineDivision of Nephrology and hypertensionMayo Clinic College of Medicine, Mayo Graduate SchoolRochesterMNUSA

**Keywords:** Signalling pathways, hypoxia, renal fibrosis

## Abstract

Renal fibrosis is the common pathological hallmark of progressive chronic kidney disease (CKD) with diverse aetiologies. Recent researches have highlighted the critical role of hypoxia during the development of renal fibrosis as a final common pathway in end‐stage kidney disease (ESKD), which joints the scientist's attention recently to exploit the molecular mechanism underlying hypoxia‐induced renal fibrogenesis. The scaring formation is a multilayered cellular response and involves the regulation of multiple hypoxia‐inducible signalling pathways and complex interactive networks. Therefore, this review will focus on the signalling pathways involved in hypoxia‐induced pathogenesis of interstitial fibrosis, including pathways mediated by HIF, TGF‐β, Notch, PKC/ERK, PI3K/Akt, NF‐κB, Ang II/ROS and microRNAs. Roles of molecules such as IL‐6, IL‐18, KIM‐1 and ADO are also reviewed. A comprehensive understanding of the roles that these hypoxia‐responsive signalling pathways and molecules play in the context of renal fibrosis will provide a foundation towards revealing the underlying mechanisms of progression of CKD and identifying novel therapeutic targets. In the future, promising new effective therapy against hypoxic effects may be successfully translated into the clinic to alleviate renal fibrosis and inhibit the progression of CKD.

## Introduction

Renal fibrosis is the common pathological hallmark of almost all advanced kidney diseases with diverse aetiologies, and it has been shown to be the most reliable predictor of CKD progression to end‐stage renal failure (ESRD). The mechanisms of interstitial fibrosis in the progression of CKD were successively supposed to be related to proteinuria, oxygen free radicals, vasoactive substances, tubular hypertrophy, hypermetabolism and endothelial dysfunction. However, current knowledge indicates that these putative causes could not account for all aspects of progressive renal diseases completely. In 2000, Fine *et al*.^1^ proposed the chronic hypoxia hypothesis, which suggested chronic oxygen deprivation, might explain the scar formation in the tubulointerstitial compartment. During the past few decades, this fascinating hypothesis has been vigorously validated both in experimental animals and in humans, and a substantial body of evidence supports the notion of chronic tubulointerstitial hypoxia as a final common pathway leading to ESRD in various pathological conditions 2, 3, 4, 5. And yet, the molecular mechanisms underlying hypoxia driving renal fibrogenesis are not well‐elucidated and numerous regulators have been supposed to be implicated in the process directly and indirectly. A comprehensive understanding of hypoxia‐regulated signalling pathways involved in the development of chronic kidney injury will undoubtedly promote the identification of novel targets against this final common pathway.

This review will focus on the pathological roles of chronic hypoxia in fibrosis progression of the kidney, and specifically explore how hypoxia promotes the fibrotic response in the interstitial compartment. We review the multiple signalling pathways and molecules involved in hypoxia‐induced pathogenesis of interstitial fibrosis, and describe predominant roles of these intracellular cascades and their interactions, in an attempt to unveil the underlying mechanisms of hypoxia‐driven renal fibrosis and provide a new insight into anti‐fibrotic strategies to alleviate renal fibrosis to halt or retard the CKD progression.

## Hypoxia promotes tubulointerstitial fibrosis

Oxygen tension is maintained by the balance between oxygen supply and oxygen consumption. Chronic oxygen deprivation in CKD actually takes place *via* multiple mechanisms when the balance is broken, ranging from decreases in oxygen supply due to glomerular damage, imbalance of vasoactive substances, peritubular capillaries rarefaction, to increases in oxygen consumption. Together, these mechanisms act at various points in concert to result in chronic hypoxia of the kidney 2, 6, 7, 8, 9. Tubular epithelial cells (TECs) are rendered particularly prone to hypoxic injury due to its high metabolic activity and large oxygen demand 10, 11, 12. Following persistent injury, the epithelial cells initiate inflammatory response by recruiting inflammatory cells to the injured interstitium and secreting a variety of fibrogenic cytokines and inflammatory factors, such as platelet‐derived growth factor (PDGF), fibroblast growth factor‐2, tumour necrosis factor‐1α (TNF‐1α) and interleukin‐6 (IL‐6), which subsequently activate fibroblasts and TECs. Activated fibroblasts are described as myofibroblasts which are principally responsible for production of extracellular matrix proteins (ECM) by regulating expression of ECM modifying factors. Furthermore, the epithelial cells are stimulated to undergo apoptosis, cell cycle arrest, and phenotypic transition as epithelial‐to‐mesenchymal transition (EMT) *via* loss of their epithelial feature and acquisition of mesenchymal phenotype, contributing to tubular atrophy and ECM accumulation. Eventually, the excess deposition of ECM in interstitium extends distance between the capillaries and nearby nephrons, and then leads to endothelial dysfunction and peritubular microvascular rarefaction, this, in turn, aggravating hypoxia and forming a vicious circle. Together, these fibrogenic events conjunctly result in tissue destruction 13, 14, 15, 16, 17, 18, 19, 20, 21, 22. Renal fibrogenesis is a complex and dynamic process involved in almost all types of renal cells, during which myofibroblasts are considered as the determining cells. The cellular origin of interstitial myofibroblasts has been in dispute, with manifold contributors proposed, such as resident fibroblasts, bone marrow‐derived fibrocytes, macrophage (MMT), pericyte and endothelial cells (EndoMT), as well as epithelial cells (EMT) 23, 24, 25, 26, 27, 28, 29. Although lineage tracing studies have doubt the existence of EMT and its contribution to the myofibroblasts pool, developing evidence suggests that EMT programme seems to undergo an incomplete process, and such a partial EMT can arrest cell cycle and thereafter halt renal repair, which leads to tissue dysfunction. In line with this notion, inhibition of EMT has been shown to reverse renal inflammation and fibrosis, indicating the crucial role of EMT in the development of renal fibrosis 30, 31, 32, 33, 34.

## Signalling pathways involved in hypoxia‐induced renal fibrosis

Renal fibrosis is a multifaceted, multilayered cellular response, and multiple signalling pathways can be activated in the hypoxic and fibrotic microenvironment. Based on recent literatures, the most important signal molecules are HIF, TGF‐β, Notch, PKC/ERK, PI3K/Akt, NF‐κb, Ang II/ROS, microRNAs, ADO, IL‐6, IL‐18 and KIM‐1. Additionally, it is apparent that these signalling pathways cooperate in the execution of scar formation, through enhancing fibroblast proliferation, activation and matrix accumulation (Tables 1 and 2). A comprehensive understanding of these cellular signalling pathways and crosstalks among them in regulating hypoxia‐induced tubulointerstitial fibrosis and kidney dysfunction is indispensable and pivotal.

**Table 1:** Signalling pathways mediated hypoxia‐induced fibrogenic responses in CKD

Signalling pathways	Related regulators	Fibrogenic effects	Ref(s)
HIF	Twist, Bmi1, LOXs, PAI1, ET‐1, TIMP‐1, MMP‐2, CTGF, VEGF, TGF‐β	Fibroblast activation, inflammatory responses, matrix modifying, collagen synthesis, EMT regulation,	33, 34, 42, 46, 47, 56
TGF‐β	Smads, TIMPs, MMPs, PHD/HIF, mTORC1, mTORC2, AngII, CTGF, ET‐1, VEGF, ILK	Fibroblast activation, inflammatory responses, matrix assembly, collagen synthesis, EMT regulation, cellular apoptosis	49, 51, 52, 55
Notch	HIF, LOXs, Snail, Hes1	Collagen synthesis, EMT regulation	48, 62, 63
NF‐κB	IL‐6, IL‐8, TNF‐α, MIP‐1, MCP‐1, iNOS, Snail, HIF, CEBPD	Inflammatory responses, EMT regulation, oxidative stress	64, 65, 66, 67
PKC/ERK	Egr‐1, Snail, TGF‐β, NF‐κB	Inflammatory responses, collagen synthesis, EMT regulation, cellular apoptosis	68, 69
PI3K/Akt	Bmi1, BVR, GSK‐3β, Snail,	Fibroblast activation, collagen synthesis, EMT regulation,	46, 73
URG11/β‐catenin	TCF, Snail, Twist, Fsp1, PAI‐1, MMP‐7	Fibroblast activation, inflammatory responses, matrix assembly, EMT regulation	80, 81, 82
Ang II/ROS	PHD/HIF, TIMP‐1, ASK1, p38/JNK, TGF‐β, PDGF‐B, PAI‐1, NF‐κB	Fibroblast activation, inflammatory responses, oxidative stress, cellular apoptosis	61, 86, 87

VEGF: vascular endothelial growth factor; Fsp1: fibroblast‐specific protein1; ILK: integrin‐linked kinase; iNOS: inducible nitric oxide synthase; ASK1: apoptosis signal‐regulating kinase 1; JNK: c‐jun N‐terminal kinase.

**Table 2:** Molecules mediated hypoxia‐induced fibrogenic responses in CKD

Molecules	Related regulators	Fibrogenic effects	Ref(s)
miR‐124,miR‐34a, miR‐155	MMP2, Notch, Snail, HIF, TGF‐β1	Fibroblast activation, inflammatory responses, EMT regulation	76, 78, 79
IL‐18	AP‐1,TLRs, TNF‐α, MIP‐2,MCP‐1STAT3	Inflammatory responses, EMT regulation, cellular apoptosis	89, 92
KIM‐1	IL‐6, MCP‐1	Inflammatory responses	93, 95
ADO	A2BR, IL‐6, PAI‐1	Fibroblast activation, matrix modifying, collagen synthesis	97, 98, 99

### HIF pathway

Hypoxia‐inducible factor (HIF) is a well‐known master mediator of hypoxia‐adaptive responses in a variety of pathophysiological processes in the kidney diseases 35, 36. Structurally, HIF belongs to basic helix–loop–helix transcription factors and consists of a hypoxically inducible α‐subunit and a constitutively expressed nuclear β‐subunit, and mammalian genomes contain three genes subtypes of HIF (HIF1‐3). Under normoxia, the conserved proline residues of HIF‐α subunits are hydroxylated by HIF‐prolyl hydroxylase domain containing proteins (PHDs). Then, hydroxylated HIF‐α can be recognized by von Hippel–Lindau protein (pVHL) which serves as the substrate recognition component of the ubiquitin ligase complex, and ultimately degraded rapidly by inducing the α‐subunit to undergo E3 ubiquitination. In hypoxic condition, the enzymatic activities of PHDs are inhibited, thereby the unmodified HIF‐α escapes from entering the destructive process but rather forms a functional complex with HIF‐β and its transcriptional co‐activators such as CBP/p300. The complex binds to hypoxia‐responsive elements (HRE) and transcriptionally regulates numerous HIF‐regulated genes, which gives rise to a number of compensatory responses against hypoxia at both cellular and physiological levels in a co‐ordinated manner 36, 37.

**Figure 1:**
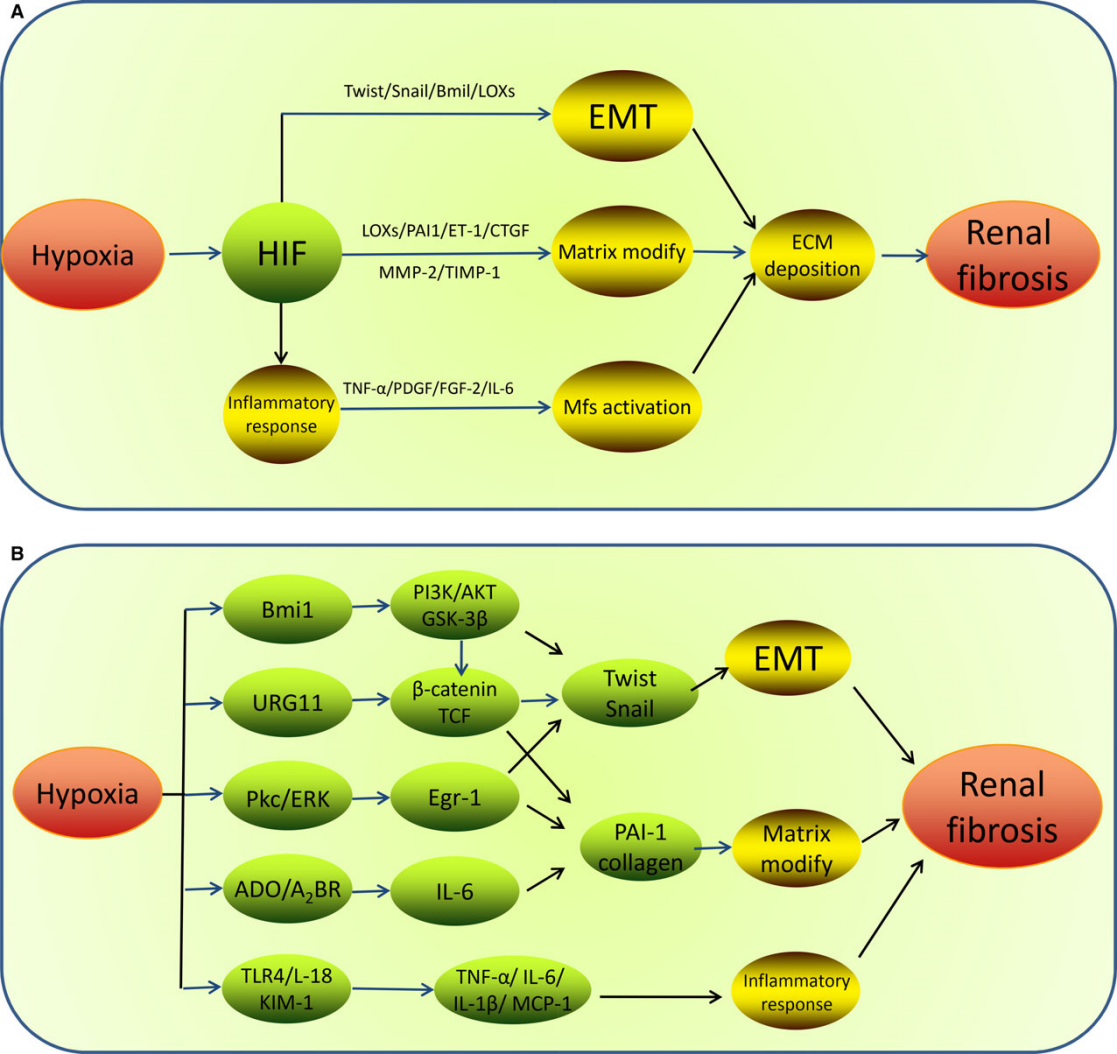
A schematic of tubulointerstitial fibrosis mediated by hypoxia‐inducible signalling pathways. (**A**) Under hypoxia condition, HIF signalling promotes renal fibrogenesis by activation of inflammatory responses, ECM, and up‐regulation of expression of EMT regulators to enhance tubular EMT, such as Twist, Bmi1, LOXs. (**B**) Additionally, other signalling pathways mediated by TGF‐β, Notch, PKC/ERK, PI3K/Akt, NF‐κB, Ang II/ROS, ADO, microRNAs, IL‐6, IL‐18, KIM‐1 and their downstream signals are also involved in hypoxia‐induced renal fibrosis, through their specific roles, respectively. EMT, epithelial‐to‐mesenchymal transition; ECM, extracellular matrix; Mfs, myofibroblasts;PAI, plasminogen activator inhibitor 1; ET‐1, endothelin‐1; CTGF, connective tissue growth factor; TIMP1, tissue inhibitor of metalloproteinase 1; TNF‐1α, tumour necrosis factor‐1α; PDGF, platelet‐derived growth factor; IL‐6, interleukin‐6; FGF, fibroblast growth factor‐2; MCP‐1, monocyte chemotactic protein‐1.

There is controversy regarding the effect of HIF on CKD pathophysiology, which seems to depend on the pathological context. On the one hand, stimulation of HIF and HIF‐regulated genes by CoCl2 has been shown to exert renoprotective role in the hypoxic tubulointerstitium in rats with nephritis 38 and hypertensive type 2 diabetes 39. A similar effect of HIF has been previously reported in cisplatin nephrotoxicity 40. Besides, there have been studies demonstrated that pharmacological activation of HIF could attenuate renal injury using the rat remnant kidney model of CKD 41, 42, 43. On the other hand, an inappropriate and prolonged activation of HIF is well known to play a pivotal role in initiating and promoting renal fibrogenesis *via* regulation of multiple signalling pathways in CKD 44, 45, 46 (Fig. 1). Firstly, HIF activation can stimulate inflammatory cells proliferation and recruitment to the site of injury in experimental models of CKD, which plays a role in setting up the fibrous scar formation. In addition, activated HIF binds to its pro‐fibrogenic downstream genes and induces maladaptive expression of matrix modifying factors directly in hypoxic TECs, such as collagen I, plasminogen activator inhibitor 1 (PAI1), endothelin‐1 (ET‐1), connective tissue growth factor (CTGF), matrix metallopeptidase 2 (MMP‐2) and tissue inhibitor of metalloproteinase 1 (TIMP1), which lead to increased production of interstitial collagen and decreased degradation of ECM. Thus, accumulating ECM components subsequently replaces the normal nephrons and fibrosis eventually occurs 47, 48, 49. Apart from promoting ECM deposition, HIF signalling is also involved in facilitating tubular EMT through modulating the expression of EMT regulators such as Snail, Slug, Zeb, SIP1, E12, FOX and CTGF, which enable TECs to lose the expression of epithelial signature and acquire mesenchymal signature to produce ECM 50, 51, 52, 53, 54. Among these EMT regulators, Sun *et al*. demonstrated that Twist expression played a crucial role in hypoxia‐regulated EMT in renal fibrosis through HIF‐1α‐dependent signalling 55, 56, 57. It has been indicated by chromatin immunoprecipitation assays and electrophoretic mobility shift that HIF‐1 can directly bind to Twist proximal HRE at 317–312 bp in TECs and regulate its expression. Under hypoxic condition, HIF‐1 induces Twist transcriptional activation and expression, leading to prohibition of TECs differentiation, myofibroblast accumulation and EMT process in TECs, as indicated by reducing the epithelial markers such as ZO‐1 and E‐cadherin while enhancing the mesenchymal markers like vimentin and α‐smooth muscle actin (α‐SMA). Thus, HIF‐twist signalling exerts a functional role in EMT‐induced renal fibrosis under hypoxia condition. Furthermore, it was reported that, Bmi1, another EMT regulator, was also essential to Twist1‐induced repression of E‐cadherin contributing to cancer metastasis 58. Our recent study in kidney diseases has demonstrated that 59 Bmi1 was involved in hypoxia‐triggered EMT in TECs and renal fibrosis, and the region of Bmi1 promoter not only contained the potential HIF‐1α‐binding site but also the Twist‐binding site. Thus, HIF‐1 and Twist cooperatively promoted Bmi1 transcriptional activation under low oxygen, then increased the expression of Bmi1 and induced the stabilization of its downstream target genes Snail and E‐cadherin by modulating PI3K/Akt pathway, which facilitated EMT process and might explain the underlying mechanisms of chronic hypoxia‐induced renal injury by enriching the HIF‐Twist signalling. Additionally, it is well known that hypoxia can promote migration of tumour cells *via* HIF‐1‐dependent induction of its target gene lysyl oxidases (LOXs) to down‐regulate E‐cadherin expression 60, 61. Experimental studies in kidney showed that HIF‐1 might drive renal fibrosis in part through up‐regulation of LOXs expression 62. The HIF‐mediated up‐regulation of LOXs induces the expression of ECM modifying factors and enhances tubular EMT *in vivo* and *vitro*. Furthermore, genetic HIF‐1 ablation can ameliorate tubulointerstitial fibrosis by reducing extracellular matrix deposition and decreasing inflammatory cell infiltration, which is consistent with the effects caused by pharmacological inhibition of LOXs. Besides, recent reports have also indicated that the LOX‐like 2 may interact with the transcriptional repressor Snail to facilitate EMT^63^. Thus, it seems that LOXs emerge as the significant mediators in pro‐fibrotic HIF signalling pathway in renal epithelial cells, *via* up‐regulating the expression of ECM modifying factors and facilitating Snail1 activation and EMT.

**Figure 2:**
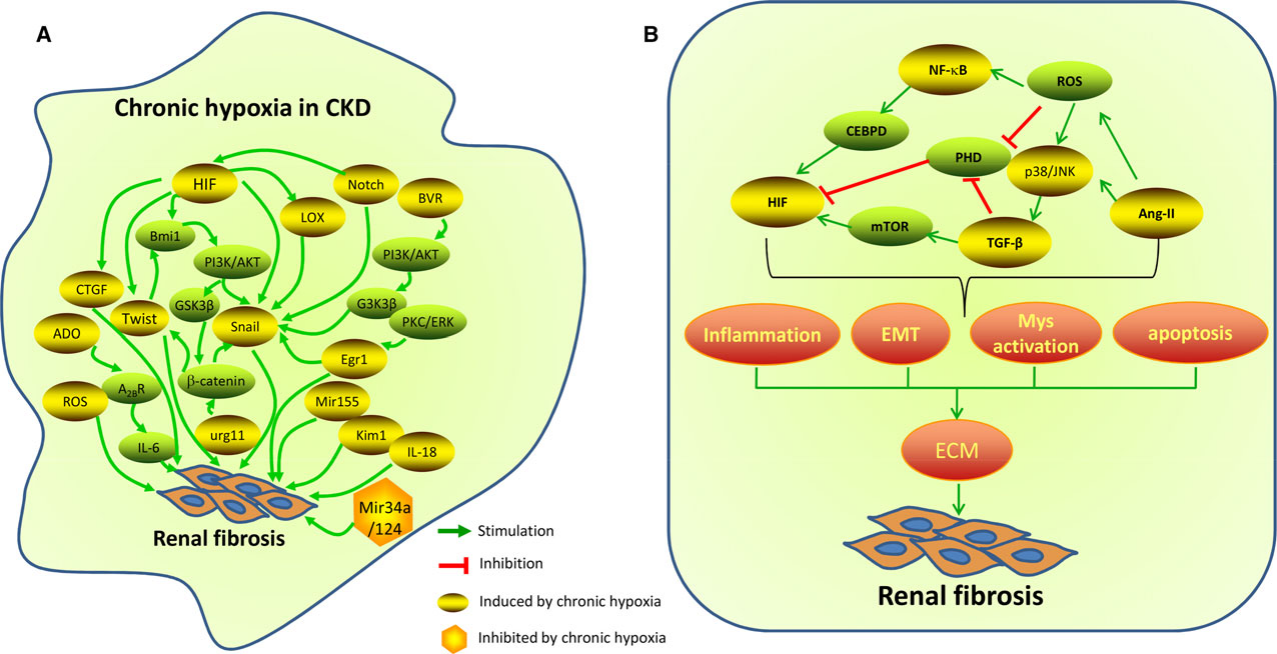
An Overview of signalling pathways(**A**) and their interactions(**B**) involved in hypoxia‐induced renal fibrosis. All these signals act at various levels in concert to amplify the pathogenesis of fibrogenic response and CKD progression. HIF, hypoxia‐inducible factor; LOXs, lysyl oxidases; CTGF, connective tissue growth factor; Egr‐1, early growth response‐1; BVR, biliverdin reductase; URG11, up‐regulated gene 11; KIM‐1, kidney injury molecule‐1; BVR, Biliverdin reductase; ADO, adenosine; IL‐6, interleukin‐6; ROS, reactive oxygen species; TGF‐β, transforming growth factor‐β; Ang II, Angiotensin II; mTOR, mammalian target of rapamycin; PHD, prolyl hydroxylase domain protein; CEBPD, CCAAT/enhancer‐binding protein δ.

In addition to hypoxic activation, it worth noting that, other factors also induce fibrogenic effects in CKD *via* oxygen‐independent activation of HIF‐1 pathway, including, for example, angiotensin II (Ang II), epidermal growth factor, TNF‐1α, interleukin‐1, nitric oxide and reactive oxygen species (ROS) 64. Furthermore, aside from its distinct fibrogenic effects, HIF signalling pathway itself might not be sufficient to promote fibrillogenesis without other cues; the crosstalks between HIF signalling pathway and other intracellular signalling pathways involved in the pathogenesis of CKD progression might be necessary to amplify the pathological fibrogenic response (see below, Fig. 2).

### TGF‐β pathway

Transforming growth factor‐β (TGF‐β) is the most ubiquitous pro‐fibrotic cytokine in progressive renal fibrosis, which signals through Smad‐dependent and non‐Smad pathways and leads to multiple downstream biological effects 65, 66, 67. In kidney fibroblasts, the expression of TGF‐β and Smad genes are increased in response to hypoxia, and subsequently activated TGF‐β‐Smads can directly stimulate myofibroblast to produce ECM proteins by increasing collagen gene expression and TIMPs but inhibiting MMPs. Meanwhile, TGF‐β can synergize with HIF to synthesis certain collagens. Some researchers proposed the pro‐fibrotic effect of the interaction between HIF and TGF‐β1/SMAD2/3 signalling pathway in chronic nephropathy 68. Falguni Das *et al*.^69^ demonstrated the involvement of mTORC1 in HIF‐1 expression for collagen I production in response to TGF‐β. They showed that treatment of TGF‐β induced increased mTORC1 activity in TECs, which enhanced the expression of HIF1 and subsequently increased collagen I gene transcription. Furthermore, both mTORC1 and mTORC2 signalling can mediate TGF‐β1‐induced interstitial fibrosis directly though the activation of fibroblast 70, 71, 72. Han *et al*. 73 found that TGF‐β1 could decrease the expression of PHD2 *via* an Smad2/3‐dependent mechanism, accordingly leading to HIF‐1α stabilization and up‐regulation the expression of fibrogenic genes such as collagen I and PAI expression in RECs. However, overexpression of PHD2 transgene decreased HIF‐1α expression and inhibited TGF‐β1‐regulated EMT, indicating the contribution of PHD2/HIF‐1α signalling pathway to the TGF‐β1‐induced EMT and renal fibrogenesis. Furthermore, hypoxia and TGF‐β1 synergistically induce dysregulated expression of vascular endothelial growth factor and endothelin in the injured tubules, resulting in an insufficient angiogenic response and aggravation of tubulointerstitial hypoxia. Similarly, connective tissue growth factor‐β (CTGF), a pro‐fibrogenic factor commonly induced by TGF‐β1, is also up‐regulated by hypoxic stimulation in the development of tubulointerstitial fibrosis. HIF can directly target CTGF at the transcriptional level, activated CTGF then induce its downstream signalling pathways to facilitate renal fibrosis and EMT, such as ERK signalling, NF‐κB pathway and Wnt signalling 74, 75, 76. However, CTGF is proposed to play a nephroprotective role of transient hypoxia as HIF‐1α reduces the CTGF expression in short‐term hypoxia of human proximal TECs 77. Furthermore, TGF‐β‐mediated induction of CTGF can be inhibited under hypoxic conditions. Additionally, there exist multiple interactions between TGF‐β and Ang II under hypoxic environment. As a promoter of fibrogenesis, Ang II can increase transcription and synthesis of TGF‐β directly and indirectly, further contributing to apoptosis and EMT. Many of the fibrogenic effects of Ang II are mainly mediated by the induction of TGF‐β and its downstream regulators of inflammation, apoptosis, and ECM synthesis, such as CTGF, PAI‐1 and ILK, which also can be directly induced by Ang II and mediate EMT and matrix assembly 78, 79, 80.

### Notch pathway

It is well‐established that Notch signalling plays an active role in EMT‐induced organ fibrosis and cancer progression under hypoxia 63, 81. Notch pathway contains four receptors (Notch1‐4) and two types of ligands, Jagged and Delta. When cells experience pathophysiological stresses, the extracellular domain of Notch receptor is bound to Notch ligand, which triggers two proteinase complexes to proteolytically cleave the notch intracellular domain (NIC), released NIC translocates into the nucleus and interacts with transcription cofactors 82. In kidney fibrosis, Notch regulates the hypoxia‐induced EMT mainly by two distinct but synergistic mechanisms. First, hypoxia activates epithelial Notch pathway directly by up‐regulating the levels of NIC and its ligand expression, which leads to increased expression of fibronectin, collagen and induction in the key EMT regulator Snail followed by reduction in E‐cadherin expression. In contrast, the inhibition of Notch activation in TECs can reduce the degree of renal fibrosis**.** And second, Notch signalling indirectly controls Snail 1 expression by potentiating crosstalk with hypoxia signalling 81. The role of Lox in HIF signalling was discussed above. It appears that Notch participates in this process as well by potentiating HIF‐1 binding to the LOX promoter and elevates the hypoxia‐induced up‐regulation of LOX, which also activates the expression of Snail 1. Additionally, upon induction of the hypoxic response, cytoplasmic HIF‐1α enhances the release of NIC by increasing the activity of the proteinase complex (γ‐secretase), and finally induces the activation of the common target gene like Hes1 83.

### NF‐κB pathway

Inflammatory response after sustaining hypoxic injury is considered as a driving force in the development of kidney fibrosis as it initiates the fibrogenic stage. Nuclear factor‐κB (NF‐κB) pathway mediates inflammatory response primarily, is activated in hypoxic epithelia cells and promotes fibrosis in liver, brain and kidney tissues through regulating its target inflammatory cytokines, adhesion molecules and pro‐inflammatory enzymes 84, 85. NF‐κB also controls the expression of EMT inducers (Snail1) and enhances EMT of mammary epithelial cells 86. Under hypoxia, NF‐κB emerges as a transcriptional regulator for HIF‐1α and leads to its protein accumulation 87. For example, CCAAT/enhancer‐binding protein δ (CEBPD), an inflammatory factor, is enhanced in TECs under both acute and chronic hypoxic conditions through NF‐κB‐dependent pathways. Yamaguchi and colleagues 88, 89 suggest that NF‐kB pathway provides a significant link between hypoxic signalling and inflammation in RECs by reinforcing CEBPD‐mediated HIF pathways; that is, hypoxia and/or inflammation lead to increased NF‐κB and CEBPD activity, CEBPD then binds to the promoter in HIF‐1α and regulates HIF‐1α signalling by transcriptionally increasing its expression level, which in turn contributes to inflammatory cell infiltration and inflammatory cytokines production in the tubulointerstitial area. Thus, the NF‐kB/CEBPD/HIF‐1 pathway plays a novel role in hypoxic renal injury.

### PKC/ERK/Egr‐1 pathway

Early growth response‐1 (Egr‐1) is identified as a vital mediator of fibroblast proliferation and inflammation triggered by various irritants. Aberrant Egr‐1 expression or function is associated with scaring process in a variety of human diseases, such as pulmonary fibrosis, emphysema and systemic sclerosis 90, 91. Egr‐1 has also been found to be a hypoxia‐responsive transcription factor, and its expression was increased in the rat model of kidney fibrosis. Our previous study provided evidence for active Egr‐1 signalling in the hypoxia‐triggered EMT and fibrosis in human TECs 92. Egr‐1 activity is mainly determined by the regulation of its biosynthesis. Both *in vivo* and *in vitro* studies implied that hypoxic conditions induced the biosynthesis of Egr‐1 *via* the PKC/ERK pathway and that Egr‐1 further promoted the renal fibrosis partially through the activation of Snail, which regulated EMT programme by repression of E‐cadherin, mucin‐1, claudins and occludins. While targeting Egr‐1 expression or suppression of ERK1/2 activity can reduce cellular apoptosis and interstitial fibrosis in unilateral ureteric obstruction (UUO) rat kidneys. Furthermore, Egr‐1 can directly induce the expression of collagen gene, matrix remodelling factors and fibrogenic cytokines TGF‐β and NF‐κB, while Egr‐1 deficiency alleviates TGF‐β‐ and NF‐κB‐induced renal fibrosis and inflammation in TECs 93. Thus, the PKC/ERK/Egr‐1 pathway plays a key role in the hypoxia‐regulated EMT and ECM. In addition, adrenomedullin, activated in human TECs cultured under hypoxic conditions, was shown to be involved in this signalling mechanism as it might inhibit hypoxia‐induced EMT by increasing the expression of E‐cadherin and ZO‐1 and decreasing α‐SMA and vimentin expression resulted from inhibition of the activation of ERK 94.

### PI3K/Akt pathway

It is well known that EMT in renal TECs and renal fibrosis induced by hypoxia is closely related to the activation of the PI3K/Akt pathway, as inhibition of PI3K activation attenuated ECM accumulation while the inhibition of Akt resulted in decrease of myofibroblast markers in obstructive nephropathy 95, 96. It is found that some transactivated EMT regulators such as Bmi1 and Biliverdin reductase (BVR) participate in hypoxia‐induced tubulointerstitial fibrosis through PI3K/Akt‐dependent pathway 59, 97. BVR has recently been considered as a serine/threonine/tyrosine kinase that might activate phosphatidylinositol 3‐kinase (PI3K) and Akt 98. First, BVR expression is up‐regulated in hypoxic renal tubular cells and remnant kidney, which leads to phosphorylation of Akt. The increase level of Akt phosphorylation then induces change in phosphorylation of GSK‐3β and up‐regulation of Snail, a downstream target protein of Akt, which can induce activation of fibroblast and accumulation of matrix production in the obstructed kidneys. Furthermore, GSK‐3β also activates β‐catenin signalling and further enhances the expression of several fibrotic genes 99. In addition, the relationship between Bmi1 and activation of the PI3K/Akt pathway has been studied recently 100. It is demonstrated that Bmi1 mediates hypoxia‐induced EMT in TECs partly *via* modulation of the PI3K/Akt/GSK‐3β pathway and stabilization of Snail.

### URG11–β‐catenin pathway

Up‐regulated gene 11 (URG11), a new HBx‐up‐regulated gene, was initially found to be involved in hepatocellular carcinoma metastasis *via* regulation the transcription of β‐catenin. Our previous work 101, 102 showed that URG11 also participated in the hypoxia‐induced EMT and renal fibrosis and its overexpression was correlated with kidney failure prognosis. Chronic hypoxia stimulated URG11 expression in UUO model, which, in turn, suppressed the expression of E‐cadherin, then activated β‐catenin and enhanced its nuclear accumulation. In nuclear, β‐catenin bond to T cell factor (TCF) and stimulated the transcription of fibrosis‐related genes, such as Snail, Twist, Fsp1, PAI‐1, MMP‐7 and fibronectin, which executed their fibrotic actions by activation of inflammatory cells and myofibroblasts, promoting EMT and ECM assembly 103.

### Ang II/ROS pathway

Angiotensin II is recognized as one of the factors causing hypoxia in tubulointerstitium *via* both structural and functional changes of peritubular microvascular system, including decrease in blood flow of peritubular capillaries and inefficient use of oxygen due to oxidative stress 104, which is defined as a dysregulation of antioxidant mechanisms and overproduction of ROS. In turn, chronic renal hypoxia can activate Ang II and induce significant oxidative stress by suppressing the expression of antioxidant such as Cu/Zn‐SOD or up‐regulating the ROS production such as NADPH oxidase 105. Ang II plays a significant role in the pathophysiology of renal inflammation and fibrosis though enhancing tubular apoptosis and EMT 106. It has also been demonstrated that ROS/PHD/HIF‐1 mediates Ang II‐induced pro‐fibrotic effect in CKD independent of hypoxia 79, 107. Ang II suppressed HIF‐prolyl hydroxylases activities by increasing reactive oxygen species production (H2O2), thereby stimulating HIF‐1 accumulation, and consequently inducing TIMP‐1 and collagen I/III production in renal cells. HIF‐1α may also play a role in AngII‐mediated RTEs transdifferentiation. In addition, ANG II and oxidative stress also induce apoptosis signal‐regulating kinase 1 (ASK1) in primary tubular cells under hypoxic stress, which then activates p38/JNK signalling and promotes the development of renal inflammation, apoptosis and fibrosis, through up‐regulation of collagen I and IV and pro‐fibrotic factors such as TGF‐β1, PDGF‐B and PAI‐1 108. Both oxidative stress and Ang II can activate NF‐κB pathway and subsequently contribute to renal inflammatory injury in UUO 109. Moreover, prolonged hypoxia and oxidative stress are well known to induce endoplasmic reticulum (ER) stress and activate the unfolded protein response (UPR), thereby leading to cell apoptosis and tubular inflammation 110, 111. Collectively, the connections among HIF‐1, hypoxia, ANG II and ROS highlight the role of metabolic and apoptosis pathways in the progressive chronic renal diseases.

### miRNAs

It is evident that hypoxia regulates the biogenesis and activity of microRNAs (miRNAs), which are short non‐coding RNAs that regulate gene expression through post‐transcriptional mechanisms 112. Numerous researches have demonstrated the vital role of miRNAs in the development of renal fibrosis and EMT. For instance, miR‐124, an anti‐fibrotic factor, was shown to be associated with hypoxia‐dependent MMP2 expression in renal tubular cells. Stephanie Zell *et al*.^113^ showed that hypoxia induced a twofold up‐regulation of MMP2 expression and fivefold down‐regulation of miR‐124 compared to normoxia in TECs. Furthermore, they demonstrated *in vitro* that overexpression of miR‐124 reduces MMP2 protein level, restoring the hypoxia‐induced enhanced RPTEC migration. MMP‐2 increased migration/proliferation of TECs and enhanced macrophage infiltration by mediating TBM degradation, contributing to the induction of EMT markers and the development of renal fibrosis 114. Similarly, the regulation of miR‐34a expression was involved in hypoxia‐induced tubular EMT by targeting Notch signalling 115. Hypoxia reduced the miR‐34a expression in HK2 cells, which in turn decreased expression of E‐cadherin and improved expression of a‐SMA and vimentin, thus enhanced EMT process. In contrast, MiR‐155 was correlated with hypoxia‐associated renal fibrosis as a pro‐fibrotic cytokine. Hypoxia induced high expression of miR‐155 and promoted fibrosis in proximal tubule cells. Functional experiments further indicated that miR‐155 was positively modulated by HIF‐1α under hypoxia and the down‐regulation of miR‐155 was observed in hypoxic renal tubular cells along with HIF‐1α knockdown. Up‐regulated miR‐155 was able to promote the enhancement of α‐SMA expression and reduction of E‐cadherin expression. What is more, the data also demonstrated that miR‐155 is also capable of promoting renal fibrosis by regulating TGF‐β1 116.

### Other molecules

Several other molecules have been reported to affect the progression of hypoxia‐induced renal fibrosis. Kidney injury molecule‐1 (KIM‐1), a transmembrane tubular protein initially found in response to acute ischaemic and toxic kidney injury, has been correlated with the pathogenesis of tubulointerstitial inflammation and fibrosis in the chronic injury in CKD. Evidence from both animal models and human diseases showed that sustained hypoxic stimulation could markedly up‐regulate the expression of KIM‐1 in TECs and sequentially stimulate epithelial cells to secrete cytokines such as IL‐6 and monocyte chemotactic protein‐1 (MCP‐1), which attract inflammatory cells to the tubulointerstitial hypoxic region and enhance inflammatory effects of prolonged hypoxia on tubular cells followed by fibrotic responses 117, 118, 119.

IL‐18 is an inflammatory cytokine involved in the pathogenesis of renal ischaemia–reperfusion injury and fibrosis 120, 121. Increasing evidence demonstrates that Toll‐like receptors (TLRs) play a role *via* mediating both pro‐inflammatory and pro‐fibrotic pathways in IL‐18‐induced renal fibrosis 122. In UUO model, the downstream pro‐fibrotic effects of IL‐18 in TECs are mediated, in part, through alterations in TLR4 expression/signalling. IL‐18 up‐regulates TLR4 expression *via* activation of activating protein‐1 (AP‐1). TLR4 stimulation induces cellular changes consistent with EMT, such as high levels of a‐SMA expression and low levels of E‐cadherin expression. Furthermore, TLR4 expression is increased in the proximal tubular cells following hypoxia and activates other pro‐inflammatory cytokine and chemokine expression (TNF‐α, IL‐1, IL‐6, IL‐8, IL‐1b, MIP‐2 and MCP‐1) 123, 124. In addition, STAT3 has also been demonstrated to mediate IL‐18‐induced pro‐fibrotic response and apoptosis in obstructed kidneys 125.

Adenosine (ADO) is a hypoxia‐induced signalling molecule binding to its receptors (ARs) on cell surface. Hypoxic milieu causes extracellular ADO accumulation by repression of the equilibrative nucleoside transporters (ENTs), channels responsible for ADO uptake to be cleared, which can be also regressed by high glucose in the diabetic kidney 126, 127. In UUO model, prolonged hypoxia occurs and then enhances ADO concentration, leading to activation of the receptor A2BR, which plays a fibrotic role by increased induction of IL‐6. IL‐6 signalling serves as a pro‐fibrotic mediator facilitating collagen production in the ADO ‐induced renal injury, such as pro‐collagenI and PAI‐1, resulting in the deposition of ECM components 128, 129. In line with these observations, increasing ADO level under hypoxia has recently been found to play a role in triggering renal fibroblast activation and proliferation. Thus, ADO signalling contributes to promotion of ECM accumulation and renal interstitial fibrosis 130.

## Conclusion and perspective

This article attempts to comprehensively evaluate the available evidence on the role of hypoxia in the renal fibrogenesis with special focus on the functions and crosstalks of the signalling pathways involved in the pathogenesis of hypoxia‐induced renal fibrogenesis firstly. It appears that most signalling pathways converge on the EMT, inflammatory responses and extracellular matrix turnover. As the mechanism underlying scaring process remains vast and more complicated than we are aware of, it is uncertain which one is playing a leading role under chronic hypoxia. However, considering the fact that substantial interactions among two or more signalling pathways synergistically facilitate interstitial fibrosis, it seems that targeting multiple signalling pathways may be a logical strategy to ameliorate interstitial fibrosis. Many anti‐fibrotic agents appear to work in animal models, such as prolyl hydroxylase inhibitor, anti‐TGF‐β antibody, Ang II type‐1 receptor inhibitor, aldosterone inhibitor and recombinant human BMP‐7 131. However, the most difficult challenge ahead is to translate these promising strategies into clinical trials. Hypoxia is likely to be the key pathogenic mechanism in CKD and later ESRD. Targeting hypoxia‐mediated processes likely hold promise in devising novel interventional strategies to retard or halt unwanted renal fibrosis and improve clinical outcome in patients with CKD.

## Disclosure statement

The authors have no conflict of interest to disclose.
